# Persistence with Statins and Onset of Rheumatoid Arthritis: A Population-Based Cohort Study

**DOI:** 10.1371/journal.pmed.1000336

**Published:** 2010-09-07

**Authors:** Gabriel Chodick, Howard Amital, Yoav Shalem, Ehud Kokia, Anthony D. Heymann, Avi Porath, Varda Shalev

**Affiliations:** 1 Maccabi Healthcare Services, Tel Aviv, Israel; 2 Sackler Faculty of Medicine, Tel Aviv University, Tel Aviv, Israel; 3 Department of Medicine D, Meir Medical Center, Kefar Saba, Israel; 4 Faculty of Health Sciences, Ben Gurion University of the Negev, Israel; Leiden University Medical Centre, Netherlands

## Abstract

**Background:**

The beneficial effects of statins in rheumatoid arthritis (RA) have been suggested previously, but it is unclear whether statins may prevent its development. The aim of this retrospective cohort study was to explore whether persistent use of statins is associated with onset of RA.

**Methods and Findings:**

The computerized medical databases of a large health organization in Israel were used to identify diagnosed RA cases among adults who began statin therapy between 1998 and 2007. Persistence with statins was assessed by calculating the mean proportion of follow-up days covered (PDC) with statins for every study participant. To assess the possible effects of healthy user bias, we also examined the risk of osteoarthritis (OA), a common degenerative joint disease that is unlikely to be affected by use of statins.

A total of 211,627 and 193,770 individuals were eligible for the RA and OA cohort analyses, respectively. During the study follow-up period, there were 2,578 incident RA cases (3.07 per 1,000 person-years) and 17,878 incident OA cases (24.34 per 1,000 person-years). The crude incidence density rate of RA among nonpersistent patients (PDC level of <20%) was 51% higher (3.89 per 1,000 person-years) compared to highly persistent patients who were covered with statins for at least 80% of the follow-up period. After adjustment for potential confounders, highly persistent patients had a hazard ratio of 0.58 (95% confidence interval 0.52–0.65) for RA compared with nonpersistent patients. Larger differences were observed in younger patients and in patients initiating treatment with high efficacy statins. In the OA cohort analysis, high persistence with statins was associated only with a modest decrement in risk ratio (hazard ratio = 0.85; 0.81–0.88) compared to nonadherent patients.

**Conclusions:**

The present study demonstrates an association between persistence with statin therapy and reduced risk of developing RA. The relationship between continuation of statin use and OA onset was weak and limited to patients with short-term follow-up.

*Please see later in the article for the Editors' Summary*

## Abstract 2

In a retrospective cohort study, Gabriel Chodick and colleagues find a significant association between persistence with statin therapy and reduced risk of developing rheumatoid arthritis, but only a modest decrease in risk of osteoarthritis.

## Editors' Summary

**Background:**

The role of statins in the management of diseases that have an inflammatory component is unclear. There is some evidence that statins may have anti-inflammatory and immunumodulatory properties, demonstrated by reducing the level of C-reactive protein that may play an important role in chronic inflammatory diseases, such as rheumatoid arthritis—a chronic condition that is a major cause of disability. Some small studies have suggested a modest effect of statins in decreasing disease activity in patients with rheumatoid arthritis, but a recent larger study involving over 30,000 patients with rheumatoid arthritis showed no beneficial effect. Furthermore, it has been suggested that statins may have a role in the primary prevention of rheumatoid arthritis, but so far there has been no solid evidence base to support this hypothesis. Before statins can potentially be included in the treatment options for rheumatoid arthritis, or possibly prescribed for the prevention of this condition, there needs to be a much stronger evidence base, such as larger studies with longer follow-up periods, which clearly demonstrates any significant clinical benefits of statin use.

**Why Was This Study Done?:**

This large study (more than 200,000 patients) with a long follow-up period (average of 10 years) was conducted to discover whether there was any kind of association between persistent use of statins and the onset of rheumatoid arthritis.

**What Did the Researchers Do and Find?:**

The researchers conducted a retrospective cohort study among the members of Maccabi Healthcare Services (a health maintenance organization [HMO]) in Israel, which has 1.8-million enrollees and covers every section of the Israeli population, to identify statin users who were at least 18 years of age and did not have RA or a related disease at study entry. The cohort covered the period 1998–2007 and included members who were continuously enrolled in the HMO from 1995 to 1998. The researchers then analyzed the incidence of newly diagnosed rheumatoid arthritis, recording the date of first diagnostic codes (International Classification of Diseases, 9th revision [ICD-9]) associated with rheumatoid arthritis during the study follow-up period. To assess any potential effects of “healthy adherer” bias (good adherence to medication in patients with a chronic illness may be more likely to lead to better health and improved survival), the researchers also examined any possible association between persistent statin use and the development of osteoarthritis, a common degenerative joint disease that is unlikely to be affected by statin use.

During the study follow-up period, there were 2,578 incident cases of rheumatoid arthritis and 17,878 incident cases of osteoarthritis. The crude incidence density rate of rheumatoid arthritis among patients who did not persistently take statins was 51% higher than that of patients who used statins for at least 80% of the follow-up period. Furthermore, patients who persistently used statins had a risk ratio of 0.58 for rheumatoid arthritis compared with patients who did not persistently use statins. In the osteoarthritis cohort analysis, high persistence with statin use was associated with a modest decrement in risk ratio (0.85) compared to patients who did not persist with statins.

**What Do These Findings Mean?:**

This study suggests that there is an association between persistence with statin therapy and reduced risk of developing rheumatoid arthritis. Although the researchers took into account the possibility of healthy adherer bias (by comparing results with the osteoarthritis cohort), this study has other limitations, such as the retrospective design, and the nonrandomization of statin use, which could affect the interpretation of the results. However, the observed associations were greater than those that would be expected from methodological biases alone. Larger, systematic, controlled, prospective studies with high efficacy statins, particularly in younger adults who are at increased risk for rheumatoid arthritis, are needed to confirm these findings and to clarify the exact nature of the biological relationship between adherence to statin therapy and the incidence of rheumatoid arthritis.

**Additional Information:**

Please access these Web sites via the online version of this summary at http://dx.doi.org/10.1371/journal.pmed.1000336.

- Arthritis Research UK provides a wide range of information on arthritis research
- The American College of Rheumatology provides information on rheumatology research
- Patient information on rheumatoid arthritis is available at Patient UK
- Extensive information about statins is available at statin answers

## Introduction

Rheumatoid arthritis (RA) is a leading cause of disability that often reduces patients' quality of life and impairs their ability to work [1]. Prevalence estimates of RA worldwide indicate that the prevalence of RA range between 2.0 to 10.7 per 1,000 adults, based on the 1987 revised American College of Rheumatology (ACR) criteria [2].

RA is a chronic systemic inflammatory condition characterized by leukocyte recruitment into synovial tissue. There is growing evidence that statins have anti-inflammatory and immunumodulatory properties, demonstrated by reducing the level of C-reactive protein (CRP) that may play an important role in RA, independent of their cholesterol lowering effects [3],[4]. A modest beneficial effect of statins in RA has been demonstrated by several small randomized clinical trials and observational studies that reported on decreased disease activity [5],[6], decreased CRP levels, reduced number of swollen joints [6],[7], and improved vascular function [8],[9]. In contrast, a large US medical and pharmacy claims study [10] showed no beneficial effect for statins among 31,451 RA patients as measured by initiation and cessation of oral steroids. Consequently, there is a need for large investigations with long follow-up periods to explore whether statins can be shown not only to improve the clinical manifestation of RA, but perhaps also to relate to lower RA occurrence. In our previous studies we examined the effect of persistent use of statins on all-cause mortality [11], incident cataract [12], and age-related macular degeneration. In the present study, we evaluated the association between persistence with statins and onset of RA among a large, unselected population of statin users who were at least 18 y of age and did not have RA or a related disease, including symptomatic osteoarthritis (OA) or rheumatic fever, at study entry.

## Methods

### Study Population

We conducted a retrospective cohort study among the members of Maccabi Healthcare Services (MHS), a 1.8-million enrollee health maintenance organization (HMO) operating in Israel. According to the Israeli National Health Insurance Act, MHS may not bar any citizen who wishes to join it, and therefore every section in the Israeli population is represented in MHS. According to the most recent report of the Israel National Insurance [13], the mean age and proportion of women among MHS members (31.0 y, 48.6%) is similar to the general population (32.4 y, 48.9%). All data were obtained from MHS automated databases that have previously been described [11] and were used to elicit information on all dispensed community prescriptions, hospital discharge data, biochemistry results, using a unique nine-digit national identification number. Research ethics approval was obtained from Assuta Medical Center institutional review board.

### Study Outcome

Incident cases of RA were defined by the date of first diagnostic codes associated with RA (Rheumatoid arthritis; International Classification of Diseases, 9th revision [ICD-9] codes 714.x) during the study follow-up period. The tendency of healthier patients to be more likely to persist with preventive treatments leads to a bias that has been termed “healthy user bias” [14]. To assess the potential effects of this possibly important bias, we also examined the association between persistence with statins and OA (Osteoarthritis; ICD-9 codes 715.x), a common degenerative joint disease that is unlikely to be affected by statin use.

### Cohort Definition

The cohort covered the period 1998–2007 and included members who were continuously enrolled in the HMO from 1995 to 1998. New users of statins were identified among all MHS enrollees aged 18 y or older, who from January 1, 1998 to July 1, 2007 had at least one dispensed prescription of 3-hydroxy-3-methylgluraryl coenzyme A (HMG-CoA) reductase inhibitors (e.g., lovastatin, pravastatin, simvastatin, atorvastatin). The date of first purchased statin was defined as the index date. We only included patients who were enrolled in MHS and did not have a statin prescription at least 3 y prior to the index date. Also excluded were patients who had been diagnosed with RA, OA, or rheumatic fever, and patients who had any dispensed relevant medications (methotrexate, sulphasalzine, prednisone, leflunomide, azathioprine, infliximab, etanercept, adalimumab, hydroxychloroquine, auranofin, rituximab [abatecept is currently not available in Israel]) prior to the index date or within 1 y after the index date.

### Proportion of Days Covered

Following previous studies [15]–[17], we calculated the mean proportion of days covered (PDC) by dividing the quantity of statins dispensed by the total time interval from index date to first diagnosis of RA or OA, death, leaving MHS, or December 31, 2007, whichever occurred first.

### Other Study Variables

Demographic variables at index date included baseline values of age, gender, marital status, place of residency, years of stay in Israel (for new immigrants). Socioeconomic level was categorized into quintiles according to the poverty index of the member's enumeration area, as defined by 1995 national census. The poverty index is based on several parameters including, household income, educational qualifications, crowding, material conditions, and car ownership [18]. Study subjects' electronic medical records were reviewed for a diagnosis of chronic conditions defined by ICD-9 codes. Diabetes mellitus patients were identified by using the MHS computerized diabetes mellitus patient registry [19]. Information on cancer history was provided by the Israel National Cancer Registry (INCR), which has collected information of diagnosed cancer cases from all medical institutions in Israel since 1960.

Information on health services utilization, such as number of hospitalizations in general hospitals, visits to outpatient clinics, and filled prescriptions of antihypertensive drugs and diuretics, was based on data collected for the year prior to the index date. Laboratory test results included liver function and the median of all low-density lipoprotein (LDL)-cholesterol tests during the year prior to the index date, as well as presence of rheumatoid factor (RF) in patients diagnosed with RA.

### Lipid-Lowering Pharmacotherapy

On the basis of previous clinical trials [20]–[24], statin therapy was categorized into three relative efficacy levels that were created from expected amounts of LDL-cholesterol reduction from baseline: (a) low efficacy (≤30% LDL reduction): daily dose of fluvastatin ≤40 mg, pravastatin ≤40 mg, simvastatin ≤10 mg, cerivastatin 0.2 mg, or lovastatin ≤40 mg or 10 mg twice daily; (b) moderate efficacy (31%–40% LDL reduction): daily dose of fluvastatin 80 mg, cerivastatin 0.3 mg or 0.4 mg, rosuvastatin <10 mg, simvastatin 20 mg or 40 mg, atorvastatin 10 mg; or (c) high efficacy (≥41% LDL reduction): simvastatin 80 mg, atorvastatin ≥20 mg, rosuvastatin ≥10 mg, pravastatin 80 mg, or lovastatin 80 mg).

### Statistical Analysis

A chi-square test for categorical variables and a Kruskal-Wallis test for continuous variables were performed to determine significant differences in baseline characteristics and levels of PDC with statins that were categorized into <20% (reference category), 20%–39%, 40%–59%, 60%–79%, and 80% or above. In the primary analysis, a Cox proportional hazards model with years of follow-up as the time scale was used to estimate hazard ratios (HRs) and 95% confidence intervals (CIs) [25] and to identify variables significantly associated with increased risk of RA. In the secondary analysis, similar regression models were computed with OA as a dependent variable. In both analyses, each participant was followed from the first purchase of statin to first diagnosis of RA (or OA), leaving MHS, or December 31, 2007, whichever occurred first. The maximum follow-up was approximately 10 y. To examine a potential prevalence incidence bias, we excluded patients with less than 1 y of follow-up. In addition, we conducted sensitivity analyses limited to patients with at least 5 y of follow-up.

The full multivariate model included the following baseline values: age at baseline (in 1-y intervals), gender, marital status, nationality, socioeconomic level, presence of chronic comorbidity, utilization of health services, LDL cholesterol level, and efficacy of the initial statin therapy. Tests for trend of ordinal variables were based on the category median values. Analyses were stratified by age categories, sex, baseline LDL levels, and efficacy of initial statin therapy. A chi-square test was performed to assess heterogeneity.

## Results

The median number of health plan enrollees during the study period was more than 1.6 million, with persons aged 18 y or above accounting for 66% of the population. After applying the inclusion and exclusion criteria, a total of 211,627 individuals for the RA analysis and 193,770 individuals for the OA analysis were identified as being newly treated with statin agents during the study period. During the study follow-up period 11,692 individuals died and 3,343 left MHS.

**Figure 1:**
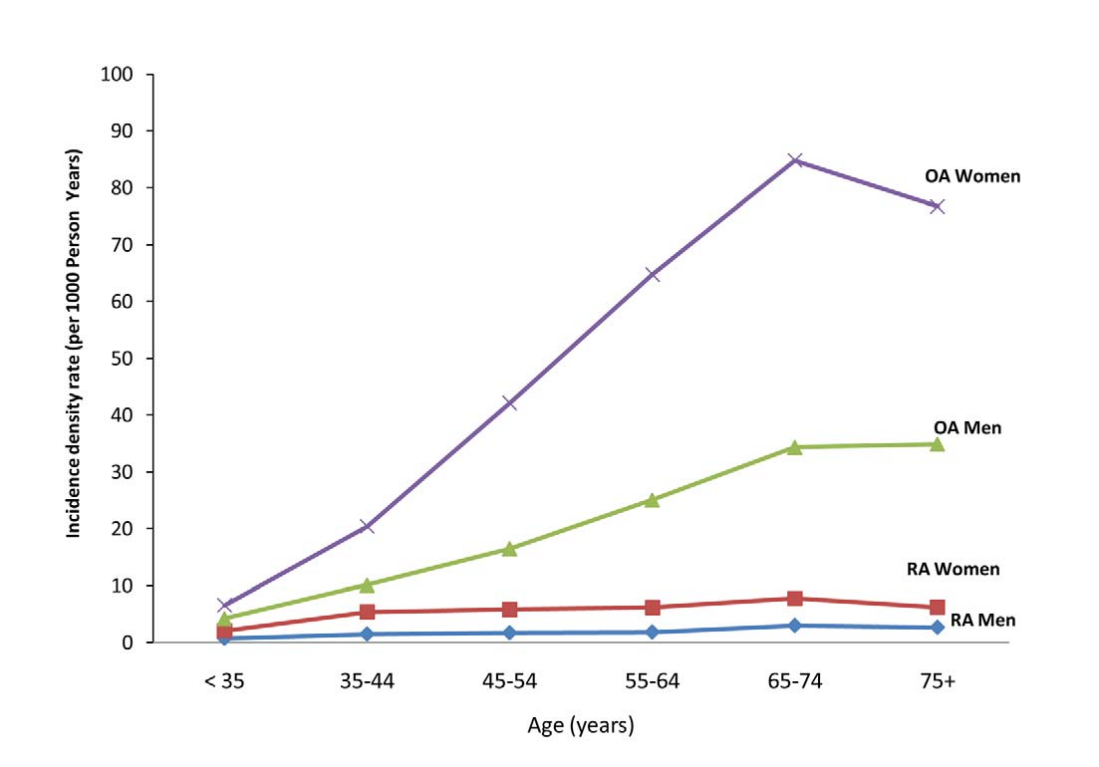
Incidence density rate (per 1,000 person-years) of RA and OA in study cohort, by age and sex, 1998–2006.

Baseline characteristics of the study population eligible for the RA analysis cohort and for the OA analysis are given in Tables 1 and 2, respectively. Patients with high PDC were more likely to be older, belong to a lower socioeconomic level, have higher prevalence of chronic conditions such as cancer, diabetes, and cardiovascular diseases, and have more frequent hospitalizations and visits to primary physicians. During 4.97 and 4.79 y of mean follow-up in the RA and OA analyses, there were 2,578 (3.07 per 1,000 person-years) RA cases and 17,878 OA cases (24.34 per 1,000 person-years), respectively. The age- and sex-specific incidence rates of RA and OA in the study population during the 10 y of study period are depicted in Figure 1. In most age groups, the incidence rate of RA and OA was 2- to 3-fold higher in women compared to men, and increased with age to a maximum in women aged 65–74 y (4.78 for RA and 50.45 per 1,000 for OA). RF tests were available for 76.6% of the diagnosed RA cases, of whom 1,478 (76.0%) were positive for RF at ≥10 IU/ml and 714 (36.7%) were positive for RF at ≥40 IU/ml.

**Table 1:** Study population characteristics, according to PDC with statins, patients eligible for the RA analysis (*n* = 211,627).

Patient Characteristics at Index Date	Substrata	Proportion of Follow-up Period Covered with Statins						*p*-Value^a^
		<20%	20%–39%	40%–59%	60%–79%	≥80%	Total	
		*n* = 57,690	*n* = 30,025	*n* = 30,542	*n* = 37,451	*n* = 55,919	*n* = 211,627	
**Age (y)**	Mean	53.55	56.52	57.70	58.51	60.07	57.17	<0.001
	(SD)	(15.13)	(12.29)	(11.82)	(11.50)	(11.09)	(12.89)	
**Sex**	Men	27,255	14,398	14,674	18,617	28,904	103,848	<0.001
	Percent	47.3	48.0	48.1	49.7	51.7	49.1	
**Socioeconomic level**	Mean	9.99	9.44	9.70	10.09	10.72	10.08	<0.001
	(SD)	(5.63)	(5.73)	(5.92)	(6.02)	(6.09)	(5.90)	
**LDL-cholesterol (mg/dl)** b	<130	11,956	4,181	4,101	5,066	8,682	33,986	<0.001
	Percent	20.7	13.9	13.4	13.5	15.5	16.1	
	130–159	14,674	8,203	8,285	10,287	16,225	57,674	
	Percent	25.4	27.3	27.1	27.5	29.0	27.3	
	160–189	15,123	9,769	10,061	12,230	17,319	64,502	
	Percent	26.2	32.5	32.9	32.7	31.0	30.5	
	≥190	7,967	5,562	5,759	6,995	8,683	34,966	
	Percent	13.8	18.5	18.9	18.7	15.5	16.5	
	Missing data	7,970	2,310	2,336	2,873	5,010	20,499	
	Percent	13.8	7.7	7.6	7.7	9.0	9.7	
**Comorbid conditions**	Obesity	9,055	5,548	5,358	6,347	9,213	35,521	<0.001
	Percent	15.7	18.5	17.5	16.9	16.5	16.8	<0.001
	Cancer	4,399	2,357	2,658	3,470	5,703	18,587	<0.001
	Percent	7.6	7.9	8.7	9.3	10.2	8.8	
	DM	10,167	8,403	8,987	11,341	17,985	56,883	
	Percent	17.6	28.0	29.4	30.3	32.2	26.9	
	CVD	17,125	10,519	12,085	16,572	28,268	84,569	
	Percent	29.7	35.0	39.6	44.2	50.6	40.0	
***n*** ** Hospitalizations** b	None	50,881	26,352	26,682	32,713	47,886	184,514	<0.001
	Percent	88.2	87.8	87.4	87.3	85.6	87.2	
	1	4,848	2,559	2,711	3,273	5,383	18,774	
	Percent	8.4	8.5	8.9	8.7	9.6	8.9	
	2 or more	1,961	1,114	1,149	1,465	2,650	8,339	
	Percent	3.4	3.7	3.8	3.9	4.7	3.9	
***n*** ** GP visits quintiles** b	≤7	15,220	6,945	6,412	7,584	10,003	46,164	<0.001
	Percent	26.6	23.2	21.1	20.3	18.0	21.9	
	8–12	12,064	6,090	6,034	7,065	9,636	40,889	
	Percent	21.1	20.4	19.8	18.9	17.3	19.4	
	13–19	11,989	6,548	6,668	8,268	12,298	45,771	
	Percent	21.0	21.9	21.9	22.1	22.1	21.7	
	20–29	9,244	5,300	5,794	7,257	11,570	39,165	
	Percent	16.2	17.7	19.1	19.4	20.8	18.6	
	30≤	8,678	5,012	5,502	7,159	12,204	38,555	
	Percent	15.2	16.8	18.1	19.2	21.9	18.3	
**Statin efficacy**	Low	22,037	9,778	10,733	13,910	22,827	79,285	<0.001
	Percent	38.2	32.6	35.1	37.1	40.8	37.5	
	Moderate	33,819	18,341	17,503	20,620	29,377	119,660	
	Percent	58.6	61.1	57.3	55.1	52.5	56.6	
	High	1,828	1,904	2,300	2,917	3,703	12,652	
	Percent	3.2	6.3	7.5	7.8	6.6	6.0	

a Kruskal-Wallis test for continuous data; χ^2^ test for categorical data.

b In the year prior to Index date.

GP, general practitioner; SD, standard deviation.

**Table 2:** Study population characteristics, according to PDC with statins, patients eligible for the OA (n = 193,770).

Patient Characteristics at Index Date	Substrata	Proportion of Follow-up Period Covered with Statins						*p*-Value^a^
		<20%	20%–39%	40%–59%	60%–79%	≥80%	Total	
		*n* = 54,378	*n* = 27,358	*n* = 27,794	*n* = 34,001	*n* = 50,239	*n* = 193,770	
**Age (y)**	Mean	52.81	55.77	57.04	57.86	59.41	56.43	<0.001
	(SD)	(15.02)	(12.20)	(11.76)	(11.46)	(11.08)	(12.87)	
**Sex**	Men	26,290	13,612	13,941	17,610	27,185	98,638	<0.001
	Percent	48.4	49.8	50.2	51.8	54.1	50.9	
**Socioeconomic level**	Mean (SD)	10.12 (5.59)	9.65 (5.70)	9.92 (5.90)	10.28 (5.99)	10.91 (6.04)	10.26 (5.86)	<0.001
**LDL-cholesterol** b **(mg/dl)**	<130	7,785	2,184	2,202	2,746	4,717	19,634	<0.001
	Percent	14.3	8.0	7.9	8.1	9.4	10.1	
	130–159	11,062	3,708	3,691	4,561	7,728	30,750	
	Percent	20.3	13.6	13.3	13.4	15.4	15.9	
	160–189	13,553	7,310	7,367	9,150	14,314	51,694	
	Percent	24.9	26.7	26.5	26.9	28.5	26.7	
	≥190	14,305	8,934	9,143	11,062	15,533	58,977	
	Percent	26.3	32.7	32.9	32.5	30.9	30.4	
	Missing data	7,673	5,222	5,391	6,482	7,947	32,715	
	Percent	14.1	19.1	19.4	19.1	15.8	16.9	
**Comorbid conditions**	Obesity	8,376	4,885	4,681	5,525	7,861	31,328	<0.001
	Percent	15.4	17.9	16.8	16.2	15.6	16.2	<0.001
	Cancer	4,038	2,095	2,404	3,092	5,070	16,699	<0.001
	Percent	7.4	7.7	8.6	9.1	10.1	8.6	
	DM	9,579	7,547	8,134	10,160	16,021	51,441	
	Percent	17.6	27.6	29.3	29.9	31.9	26.5	
	CVD	16,040	9,585	11,074	15,047	25,514	77,260	
	Percent	29.5	35.0	39.8	44.3	50.8	39.9	
***n*** ** Hospitalizations** b	None	48,080	24,076	24,319	29,722	43,039	169,236	<0.001
	Percent	88.4	88.0	87.5	87.4	85.7	87.3	
	1	4,494	2,268	2,446	2,943	4,780	16,931	
	Percent	8.3	8.3	8.8	8.7	9.5	8.7	
	2 or more	1,804	1,014	1,029	1,336	2,420	7,603	
	Percent	3.3	3.7	3.7	3.9	4.8	3.9	
***n*** ** GP** b **visits (quintiles)**	Lowest (≤7 visits)	14,909	6,656	6,177	7,284	9,514	44,540	<0.001
	Percent	27.7	24.4	22.3	21.5	19.0	23.1	
	2nd (8–12 visits)	11,648	5,775	5,635	6,724	9,078	38,860	
	Percent	21.6	21.2	20.4	19.8	18.1	20.2	
	3rd (13–19 visits)	11,348	6,020	6,164	7,615	11,309	42,456	
	Percent	21.1	22.1	22.3	22.5	22.6	22.0	
	4th (20–29 visits)	8,511	4,671	5,196	6,451	10,215	35,044	
	Percent	15.8	17.2	18.8	19.0	20.4	18.2	
	Highest (≥30 visits)	7,474	4,108	4,490	5,813	9,918	31,803	
	Percent	13.9	15.1	16.2	17.2	19.8	16.5	
**Statin efficacy**	Low	20,712	8,886	9,730	12,575	20,484	72,387	<0.001
	Percent	38.3	32.7	35.2	37.2	41.0	37.6	
	Moderate	31,498	16,528	15,738	18,517	26,029	108,310	
	Percent	58.3	60.8	56.9	54.8	52.1	56.2	
	High	1,804	1,763	2,175	2,709	3,450	11,901	
	Percent	3.3	6.5	7.9	8.0	6.9	6.2	

a Kruskal-Wallis test for continuous data; χ^2^ test for categorical data.

b In the year prior to Index date.

CVD, cardiovascular disease; DM, diabetes mellitus; GP, general practitioner; SD, standard deviation.

The crude incidence density rate of RA among patients with a PDC level of less than 20% (3.89 per 1,000 [95% CI 3.62–4.17]) was higher by 51% compared to patients with a PDC level of 80% (2.57 per 1,000 [2.37–2.78]). No similar pattern was observed in the OA analysis where the incidence density rate of OA in the lowest PDC level category (23.61 per 1,000: 95% CI 22.92–24.31) was comparable to the incidence in the highest PDC level (24.12 per 1,000) (Table 3).

**Table 3:** Incidence density rates (IDR) of RA and OA according to the PDC with statins, MHS 1998–2007.

PDC with Statins	Follow-up Mean (SD)	Person-Years at Risk^a^	*n* Cases	IDR per 1,000	95% CI
**RA analysis**					
<20% (n = 57,690)	4.55 (2.46)	204732	796	3.89	3.62–4.17
20%–39% (n = 30,025)	4.82 (2.66)	114822	356	3.10	2.79–3.44
40%–59% (n = 30,542)	5.12 (2.69)	125806	403	3.20	2.90–3.53
60%–79% (n = 37,451)	5.21 (2.76)	157550	411	2.61	2.36–2.87
≥80% (n = 55,919)	5.26 (2.83)	238157	612	2.57	2.37–2.78
Total (n = 211,627)	4.97 (2.69)	841067	2578	3.07	2.95–3.19
**OA analysis**					
<20% (n = 54,378)	4.42 (2.42)	186120	4394	23.61	22.92–24.31
20%–39% (n = 27,358)	4.64 (2.61)	99457	2501	25.15	24.18–26.14
40%–59% (n = 27,794)	4.93 (2.65)	109269	2663	24.37	23.46–25.30
60%–79% (n = 34,001)	4.99 (2.71)	135512	3395	25.05	24.23–25.90
≥80% (n = 50,239)	5.06 (2.78)	204173	4925	24.12	23.46–24.80
Total (n = 193,770)	4.79 (2.64)	734531	17878	24.34	23.99–24.69

a Excluding first year of follow-up.

SD, standard deviation.

Baseline characteristics associated with increased risk for RA and OA included age, female gender, and frequent visits to primary physicians (Tables 4 and 5). Morbid obesity was related to a substantially higher risk of OA (HR = 1.72; 95% CI 1.65–1.78), but not to RA. Similar results were obtained when analyses were limited to patients with at least 5 y of follow-up.

**Table 4:** Mutually adjusted HRs and 95% CIs for RA according to PDC with statins and baseline characteristics, MHS 1998–2007.

Factors Associated with RA Onset	>1 y of Follow-up (2,375 Cases)			>5 y of Follow-up (892 Cases)		
	HR	95% CI	*p*-Value^a^	HR	95% CI	*p*-Value^a^
**Age (per 1 y)**	1.01	1.01–1.01	<0.001	1.01	1.01–1.02	<0.001
**Sex (women vs. men)**	1.95	1.78–2.13	<0.001	1.91	1.65–2.22	<0.001
**Socioeconomic level**	1.01	1.01–1.02	<0.001	1.02	1.01–1.03	0.001
***n*** ** GP visits quintile** b						
≤7	1 (ref.)			1 (ref.)		
8–12	0.95	0.81–1.11	0.510	0.91	0.70–1.18	0.477
13–19	1.19	1.04–1.38	0.015	1.27	1.00–1.61	0.046
20–29	1.41	1.22–1.62	<0.001	1.34	1.05–1.70	0.018
30+	1.98	1.72–2.27	<0.001	1.97	1.56–2.49	<0.001
***n*** ** Hospitalizations** b						
None	1 (ref.)			1 (ref.)		
1	0.88	0.76–1.02	0.086	0.93	0.74–1.16	0.518
2+	0.85	0.69–1.05	0.133	0.84	0.60–1.19	0.327
**Chronic conditions:**						
CVD (yes vs. no)	0.96	0.88–1.05	0.364	1.00	0.87–1.16	0.981
Morbid obesity (yes vs. no)	1.02	0.91–1.14	0.753	1.12	0.94–1.34	0.201
Cancer (yes vs. no)	0.89	0.78–1.02	0.093	0.98	0.79–1.20	0.821
Diabetes (yes vs. no)	1.02	0.93–1.11	0.737	1.03	0.89–1.19	0.683
**LDL level** b **(mg/dl)**						
<130	1 (ref.)			1 (ref.)		
130–159	0.94	0.83–1.06	0.323	0.96	0.78–1.18	0.700
160–189	0.96	0.85–1.09	0.523	0.97	0.79–1.18	0.744
190+	0.84	0.72–.97	0.014	0.97	0.77–1.21	0.765
**Statin efficacy**						
Low	1 (ref.)			1 (ref.)		
Moderate	1.08	0.99–1.18	0.082	0.96	0.83–1.11	0.606
High	1.13	0.95–1.33	0.161	1.14	0.89–1.45	0.292
**PDC with statins**						
<20%	1 (ref.)			1 (ref.)		
20%–39%	0.74	0.65–0.85	<0.001	0.75	0.60–0.94	0.014
40%–59%	0.77	0.68–0.88	<0.001	0.75	0.60–0.93	0.01
60%–79%	0.61	0.54–0.69	<0.001	0.69	0.56–0.85	<0.001
≥80%	0.58	0.52–0.65	<0.001	0.69	0.57–0.83	<0.001

a Kruskal-Wallis test for continuous data; χ^2^ test for categorical data.

b In the year prior to Index date.

CVD, cardiovascular disease; GP, general practitioner.

**Table 5:** Mutually adjusted HRs and 95% CIs for OA according to PDC with statins and baseline characteristics, MHS 1998–2007.

Factors associated with OA Onset	>1 y of Follow-up (16,595 Cases)				>5 y of Follow-up (5,285 Cases)			
	HR	95% CI		*p*-Value^a^	HR	95% CI		*p*-Value^a^
**Age (per 1 y)**	1.03	1.03	1.03	<0.001	1.03	1.02	1.03	<0.001
**Sex (women vs. men)**	1.81	1.75	1.88	<0.001	1.78	1.68	1.89	<0.001
**Socioeconomic level**	0.98	0.98	0.98	<0.001	0.98	0.98	0.99	<0.001
***n*** ** GP visits** b								
≤7	1 (ref.)				1 (ref.)			
8–12	1.08	1.02	1.15	0.006	1.10	0.99	1.22	0.079
13–19	1.24	1.18	1.31	<0.001	1.30	1.18	1.44	<0.001
20–29	1.56	1.48	1.65	<0.001	1.58	1.44	1.74	<0.001
30≤	1.95	1.85	2.06	<0.001	1.91	1.73	2.11	<0.001
***n*** ** Hospitalizations** b								
None	1 (ref.)				1 (ref.)			
1	0.90	0.85	0.95	<0.001	0.88	0.80	0.97	0.008
2+	0.79	0.73	0.86	<0.001	0.83	0.72	0.95	0.007
**Chronic conditions:**								
CVD (yes vs. no)	0.91	0.88	0.94	<0.001	0.96	0.90	1.01	0.143
Morbid obesity (yes vs. no)	1.72	1.65	1.78	<0.001	1.75	1.64	1.88	<0.001
Cancer (yes vs. no)	0.94	0.89	0.98	0.007	0.97	0.89	1.05	0.448
Diabetes (yes vs. no)	0.97	0.94	1.01	0.139	0.95	0.90	1.01	0.094
**LDL level** b **(mg/dl)**								
<130	1 (ref.)				1 (ref.)			
130–159	1.04	0.99	1.09	0.123	1.04	0.96	1.13	0.351
160–189	1.01	0.96	1.06	0.672	0.98	0.90	1.07	0.689
190+	0.95	0.90	1.00	0.075	1.04	0.95	1.14	0.443
**Statin efficacy**								
Low	1 (ref.)				1 (ref.)			
Moderate	1.05	1.02	1.09	0.002	1.05	0.98	1.11	0.148
High	1.14	1.08	1.21	<0.001	1.07	0.97	1.18	0.196
**PDC with statins**								
<20%	1 (ref.)				1 (ref.)			
20%–39%	0.93	0.88	0.98	0.005	1.08	0.98	1.19	0.113
40%–59%	0.87	0.83	0.92	<0.001	1.01	0.92	1.12	0.775
60%–79%	0.89	0.85	0.93	<0.001	1.10	1.01	1.20	0.037
≥80%	0.85	0.81	0.88	<0.001	1.06	0.97	1.15	0.188

a Kruskal-Wallis test for continuous data; χ^2^ test for categorical data.

b In the year prior to Index date.

CVD, cardiovascular disease; GP, general practitioner.

**Figure 2:**
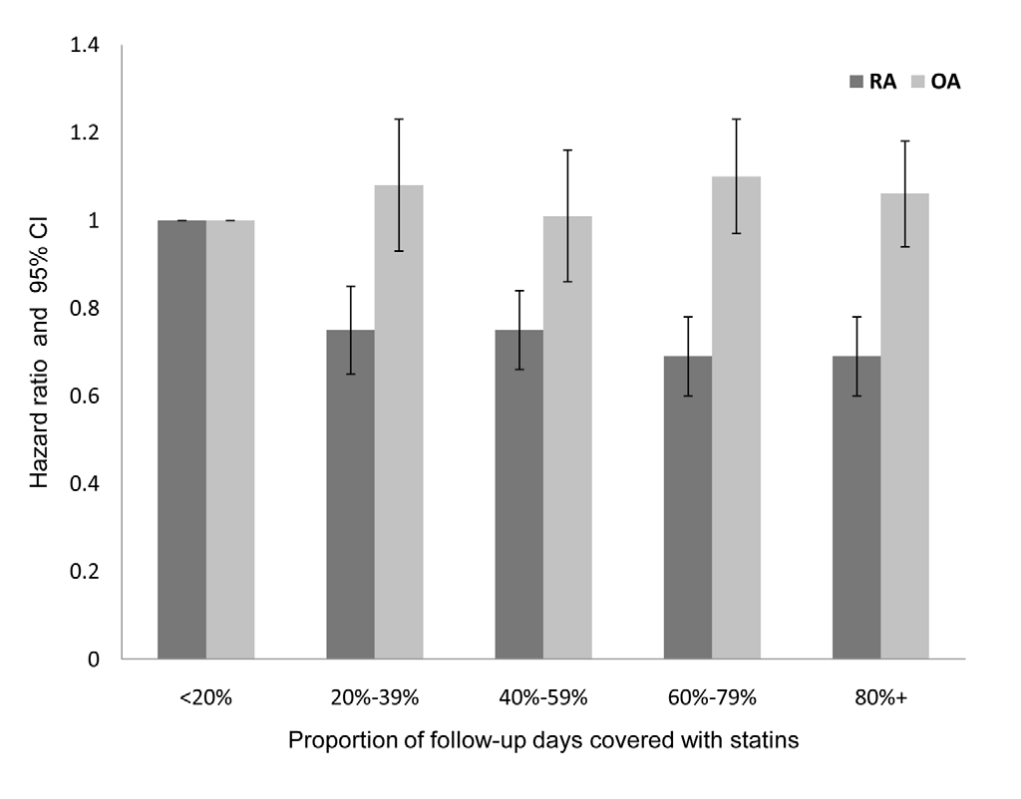
Adjusted HR and 95% CI for RA and OA, according to PDC with statins in patients with at least 5 y of follow-up. Adjusted for baseline values of age, sex, socioeconomic level, utilization of healthcare services in the year prior to index date, chronic comorbidity (cardiovascular diseases, diabetes mellitus, cancer, morbid obesity), LDL level, and statin efficacy.

After adjustment for the variables in Table 4, patients with a PDC with statins of 80% or above, had a HR of 0.58 (95% CI 0.52–0.65) for RA compared with patients with PDC levels of less than 20% (Table 4). Similar results were obtained when analyses were restricted to patients with ≥5 y of follow-up. In the OA cohort analysis (Table 5), high persistence with statins (PDC level ≥80%) was associated only with a modest decrement in risk ratio (HR = 0.85; 0.81–0.88) compared to nonadherent patients (PDC level <20%). In contrast with the RA analysis, the negative association between OA risk and PDC with statins was not observed when analyses were limited to patients with at least 5 y of follow-up (Figure 2).

**Figure 3:**
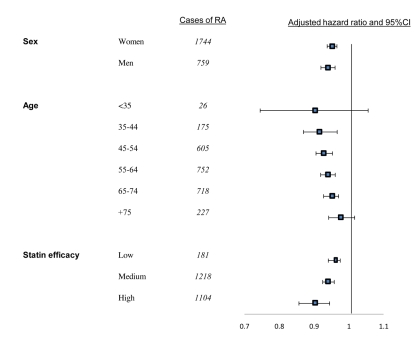
Proportional effects of persistence with statins on reduction of risk for RA per 10% of follow-up days covered with statins. Squares indicate adjusted HRs, horizontal lines, 95% CIs. Mutually adjusted for all covariates listed in Table 4.

When PDC with statins was analyzed as a continuous variable, an increase of 10% in PDC level was associated with an adjusted HR of 0.95 (95% CI 0.94–0.96) or a 5.3% lower risk of RA. Similar results were obtained when analyses were limited to patients with ≥5 y of follow-up. In the OA analysis, a 10% increase in PDC was related with an adjusted HR of 0.99 (95% CI 0.99–1.00) for OA. No association (*p* = 0.23) was calculated when analyses were limited to patients with ≥5 y of follow-up. In stratified analyses, younger age at index date was associated with larger differences in RA risk. In patients aged 35–44 y, an increase of 10% in days covered with statins was associated with an adjusted HR of 0.90 (95% CI 0.87–0.97), whereas in patients aged 75 y or above, there was no significant association between persistence with statins and reduced RA risk. However, a heterogeneity test between all age strata did not reach statistical significance. Similarly, substantially lower risk of RA was calculated for patients persistently treated with high efficacy statins with a statistically significant heterogeneity (Figure 3).

## Discussion

The present study demonstrates a significant negative association between persistence with statin therapy and RA onset, particularly in adult patients who began treatment at a relatively young age and with high efficacy statins. Our results agree with a previous nested case-control study [26] in hyperlipidemia patients, which compared 313 RA patients and 1,252 matched controls. In that analysis, the adjusted OR for development of RA in subjects taking statins compared to the reference group was 0.59 (95% CI 0.37–0.96). Similar to the present study, it was also found that patients receiving high efficacy statins (atorvastatin) had a lower odds ratio for contracting RA, although the difference did not reach statistical significance.

Several of the cholesterol-independent effects of statins are exerted by the modulation of the synthesis of isoprenoids. Post-translational modification by isoprenylation is inhibited by statins; statins decrease isoprenylation of the GTP-binding proteins Ras and Rho, which consequently leads to the modulation of signaling pathways involving endothelial nitric oxide synthase [27], tissue plasminogen activator [28], endothelin 1 [29], plasminogen activator inhibitor 1 [30], and CRP [31]. Statins were also shown to effectively suppress the induction of class II major histocompatibility complex (MHC) protein and gene expression by interferon-γ (IFNγ), resulting in suppressed class II MHC-mediated T cell activation [32]. Interestingly, statins also selectively block β2 integrin and lymphocyte function-associated antigen 1 (LFA-1). In addition LFA-1 binding to intercellular adhesion molecule 1 (ICAM-1) was also abrogated by statins [33].

In a murine model of autoimmune encephalomyelitis, atorvastatin altered cytokine secretion favoring Th2 cytokine (interleukin [IL]-4, IL-5, IL-10, and transforming growth factor β) secretion while inhibiting the expression of Th1 cytokines (IL-2, IL-12, IFNγ, and tumor necrosis factor α [TNFα]). Interestingly, CD40 signaling, which is implicated in the pathogenesis of many autoimmune conditions, was reduced by statins in atheroma-associated cells in vitro and in atherosclerotic lesions; this change hinders proinflammatory cytokines, chemokines, matrix metalloproteinases, and tissue factor secretion and activity [34].

Additional features of statins are bone-specific anabolic and antiresorptive effects that may prevent osteoporosis, which often occurs in patients with active RA. These mechanisms may elucidate the improved, yet modest overall, RA disease activity, swollen joint scores, and reduced CRP in the TARA (Trial of Atorvastatin in Rheumatoid Arthritis) trial, a 6-mo, placebo-controlled, randomized clinical trial with Atorvastatin [6].

The JUPITER (Justification for the Use of Statins in Prevention: an Intervention Trial Evaluating Rosuvastatin) trial was designed to investigate the preventive effects of Rosuvastatin against vascular events among individuals with LDL cholesterol <130 mg/dl and enhanced innate immune response, as determined by a high-sensitivity CRP (hsCRP) level. The results of the JUPITER trial indicated that statin therapy may also reduce all cause mortality in patients with low LDL cholesterol, probably by decreased inflammation [35]. Although RA patients were not included in the JUPITER trial, the questionable validity of its conclusions [36], and the uncertainty of whether or not CRP itself is a marker of risk or the target for therapy, our findings may support the potential relationship between statin use and RA risk [37].

The strengths of the current study include prospective data collection, the use of administrative databases to avoid the problem of differential recall bias, the systematic and comprehensive collection of personal sociodemographic data, medical history, and utilization of health services prior to the index date, which reduces the possibility for bias due to study outcomes. The present retrospective cohort is one of the largest undertaken to date on the relationship between statin therapy and RA, with respect to the length of follow-up and the size of the study population. Furthermore, survivor-treatment selection bias and competing medical issues, two potential problems with observational studies of treatment efficacy [38], are unlikely to affect our results, since the association of statins and RA onset was not attenuated when analyses were limited only to patients with more than 5 y of follow-up data.

In addition, the present study used internal comparisons among patients who had at least one dispensed prescription of statins, reducing the threat of confounding by indication that might have occurred in previous investigations that compared statin users and nonusers [26]. For example, a recently published cohort study [39] on more than 2 million patients from 368 general practices in England and Wales found no significant association between statin use and RA. In their analysis, Hippisley-Cox and Coupland compared the risk of RA in statin users and nonusers. The study groups differed considerably in many important characteristics such as mean age (57 versus 44 y), body mass index (BMI) (28.3 versus 26), and potentially in other important variables that were not included in the multivariable analysis (e.g., cholesterol level, LDL levels, cardiovascular diseases, and other comorbid conditions, etc.). In addition, the authors reduced the statistical power of their tests by stratifying the analyses by sex and five types of statins, and did not take into account the effect of time and amount of statin purchased. All of the above mentioned aspects could have masked a significant association between persistent use of statins and RA.

This study has the following limitations. Our analysis was retrospective in nature and allocation of statin therapy was not randomized. Despite adjustment for baseline differences and an abundance of poor prognostic factors, a higher proportion of days covered with statins could still be a surrogate for other unmeasured variables that reflect a higher quality of care and more aggressive treatment strategies. In our analysis we did not address different temporal variations in patients' use of statins over the study period. However, a majority of RA patients (79%) purchased statins at least 3 mo prior to diagnosis. Also, the similarity in study results when limiting the analysis to patients with at least 5 y of follow-up reduces the possible effect of this potential limitation.

The most important methodological limitation in estimating effectiveness in observational studies is the potential for “healthy adherer” bias. Previous studies have indicated that in patients with a chronic illness, good adherence to medication, or even to placebo, is more likely to lead to better health and improved survival [40]. A recent study [41] aimed to examine whether adherence with statins is associated with a decreased risk of accidental events that were thought to be unrelated to statins (e.g., motor vehicle and workplace accidents, burns, and falls). As expected, they found a modest (10%–15%) overall reduction in accident rate among adherent patients compared to nonadherent ones. In order to evaluate this potentially important bias in our study, we conducted a similar analysis with OA as an outcome. Our results indicated that persistent use of statins was associated with a 15% lower OA risk, a relatively small difference compared to RA risk, but one that needs to be noted when considering the results of the study overall. In addition, the reduced risk for OA in patients with high PDC with statins was limited to patients with short follow-up periods and was not found in patients with a follow-up of 5 y or more. This finding supports the notion that most of the RA risk reduction is due to a real biological effect. The threat of “healthy adherer bias” in this study was further diminished by findings from our previous study [42] on the present cohort indicating that older age and the presence of chronic diseases and other risk factors for cardiovascular events are associated with higher persistence with statins.

The incidence of RA and OA in our study, based on physician diagnoses, is higher compared with previous studies [2],[43],[44]. This higher incidence can be explained by the relatively older age and the frequent visits to physicians in our study population, leading to earlier detection. Also, RA cases in most epidemiological studies were defined by the 1987 ACR criteria and not on diagnosis alone as in our study. The association between LDL levels and risk of RA is not fully understood. Some studies [45]–[47], but not all [48], suggested that patients with active untreated RA have reduced LDL levels. A recent large retrospective cohort from the Rochester Epidemiology Project showed a decrement in LDL levels during the 5-y period before RA incidence. This decrement could not be explained by usage of lipid-lowering drugs alone. This conclusion is in agreement with our finding that high LDL levels (>190 mg/dl) at index date were significantly associated with a lower short-term (<5 y) risk of RA. Since patients with higher LDL levels at index date are more likely to be persistent with statin therapy [49], a residual confounding cannot be excluded.

Mild muscle pains are one of the frequent side effects of statins documented in 5% to 10% of outpatients on statins [50],[51] and commonly result in discontinuation of treatment. Muscle symptoms frequently begin within several months after initiation of therapy [50]. Since muscle pains can be misclassified as OA symptoms and result in result in a mistaken diagnosis of OA shortly after treatment initiation. This potential differential information bias may explain the disappearance of the small negative association between statin use and OA when analysis was limited to patients with at least 5 y of follow-up. Also, one might claim that persistent use of statins may have been associated with more frequent physician visits increasing the chance of being diagnosed with RA or OA. However, the direction of this potential detection bias may only support our conclusions. In the present research, not only did the risk of RA not rise with increasing persistence, it decreased, indicating that the true association between persistent statin use and RA could be stronger than observed. Interestingly, starting statin treatment at a younger age (35–44 y) was associated with a more pronounced decline of onset of RA; this finding probably underlines the importance of inflammatory processes during this age and the potential role statins may have to block these mechanisms, in other words, the different effects that statins have at different ages once again implies that RA is a heterogeneous disease.

In conclusion, this study showed that persistence with statin treatment was associated with an ongoing decrement in the risk for contracting RA. The observed associations were greater than those that would be expected from methodological biases alone. Larger, systematic, controlled, prospective studies with high efficacy statins, particularly in younger adults who are at increased risk for RA, are needed to confirm our findings, and to elucidate the possible biological relationship between adherence to statin therapy and RA onset.

## Abbreviations

CI: confidence interval
CRP: C-reactive protein
HR: hazard ratio
LDL: low-density lipoprotein
MHS: Maccabi Healthcare Services
OA: osteoarthritis
PDC: proportion of follow-up days covered
RA: rheumatoid arthritis
RF: rheumatoid factor
